# Non-canonical regulation of phosphatidylinositol 3-kinase gamma isoform activity in retinal rod photoreceptor cells

**DOI:** 10.1186/s12964-015-0087-9

**Published:** 2015-02-03

**Authors:** Vivek K Gupta, Ammaji Rajala, Raju VS Rajala

**Affiliations:** Department of Ophthalmology, University of Oklahoma Health Sciences Center, 608 Stanton L. Young Boulevard, Oklahoma City, OK 73104 USA; Department of Physiology, University of Oklahoma Health Sciences Center, 608 Stanton L. Young Boulevard, Oklahoma City, OK 73104 USA; Department of Cell Biology, University of Oklahoma Health Sciences Center, 608 Stanton L. Young Boulevard, Oklahoma City, OK 73104 USA; Dean A. McGee Eye Institute, 608 Stanton L. Young Boulevard, Oklahoma City, OK 73104 USA

**Keywords:** Cyclic nucleotide-gated channel, Phosphatidylinositol 3-kinase gamma, Photoreceptor outer segments, Ras-associating domain, Ras-like domain

## Abstract

**Background:**

Phosphatidylinositol 3-Kinases (PI3Ks) are a family of lipid kinases that phosphorylate the D3-hydroxyls of the inositol ring of phosphoinositides, and are responsible for coordinating a diverse range of cellular functions. A canonical pathway of activation of PI3Ks through the interaction of RA-domain with Ras proteins has been well established. In retinal photoreceptors, we have identified a non-canonical pathway of PI3Kγ activation through the interaction of its RA-domain with a putative Ras-like domain (RLD) in alpha subunit of cyclic nucleotide-gated channel (CNGA1) in retinal rod photoreceptors.

**Results:**

The interaction between PI3Kγ and CNGA1 does not appear to play a role in regulation of CNG channel activity, but PI3Kγ uses CNGA1 as an anchoring module to achieve close proximity to its substrate to generate D3-phosphoinositides.

**Conclusions:**

Our studies suggest a functional non-canonical PI3Kγ activation in retinal rod photoreceptor cells.

## Introduction

Phosphatidylinositol 3-Kinases (PI3Ks) are a family of lipid kinases that catalyze the phosphorylation of D3-hydroxyls in the inositol head group and generate several phosphorylated phosphoinositides [1]. The D3-phosphorylated products further act as membrane tethers to several phospholipid binding proteins which regulate a diverse range of cell functions, including proliferation, cell survival, degranulation, vesicular trafficking, and cell migration [1]. There is a highly conserved Ras-associating (RA) domain present in different isoforms of class I and class II PI3Ks [1]. A canonical pathway of the activation of PI3K through interaction of its RA-domain with Ras protein has been well established [2]. In retinal photoreceptors, we found a non-canonical pathway of PI3Kγ activation through interaction of its RA-domain with a putative Ras-like domain (RLD) in the cyclic nucleotide-gated channel alpha subunit (CNGA1) in retinal rod photoreceptors.

Photoreceptor cyclic nucleotide-gated (CNG) channels are critical elements in phototransduction and light adaptation [3,4]. They are responsible for generating the light response in photoreceptors and are directly and co-operatively gated by the availability of cGMP [3]. Interestingly, PI3K-generated phosphoinositides have also been shown to modulate the olfactory [5,6] and cone photoreceptor CNG [7] channels. However, the interaction between PI3Kγ and CNGA1 does not appear to play a role in regulation of CNG channel activity.

This first report of PI3Kγ interaction with a Ras-like domain in the nucleotide binding region of CNGA1 provides a framework within which detailed kinetic and structural studies can be carried out to establish the detailed mechanism and functional relevance of this interaction. The functional regulation of PI3Kγ by G-protein coupled receptors [8], Ras [2], and CNG channels through its RA domain establishes the important position of PI3Ks in signal transduction and provides further evidence of how closely the cell signaling network is integrated with phototransduction.

## Results

### PI3K interacts with CNGA1

We previously reported that the C-terminal region of CNGA1 displays 50-70% tertiary structural similarity towards Ras proteins [9]. We named this region the Ras-like (RLD) domain. Our studies also suggest that growth factor receptor-bound protein 14 (Grb14), a Ras-associating (RA) domain-containing protein, binds to CNGA1 and modulates channel activity [10]. Our membrane yeast two-hybrid screens also identified PI3K as a CNGA1 interaction partner (data not shown).

**Figure 1:**
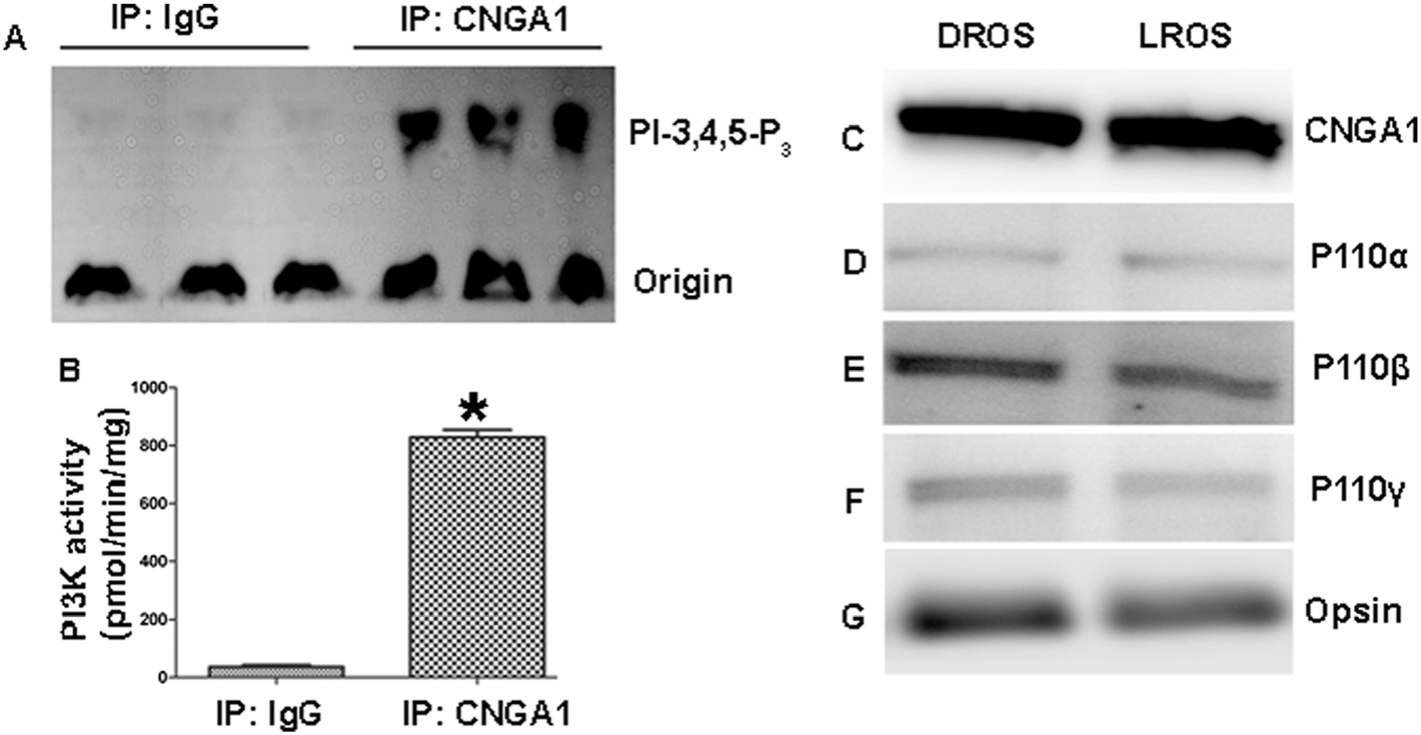
**PI3K interacts with CNGA1** ***in vivo.*** PI3K enzyme activity was measured in anti-CNGA1 or non-immune IgG immunoprecipitates from solubilized ROS. PI3K activity was measured using PI-4,5-P_2_ and [γ^32^P]ATP as substrates **(A)**. The radioactive spots of PI-3,4,5-P_3_ were scraped from the TLC plate and counted **(B)**. Data are mean ± *SD* (*n* = 3), *p < 0.001. Immunoblot analysis of ROS proteins from dark- and light-adapted rats with anti-CNGA1**(C)**, p110α **(D)**, p110β **(E)**, p110γ **(F)**, and anti-opsin **(G)** antibodies.

The RA-domain is present in class IA (p110α, β, δ), class IB (p110 γ), and class II (PI3K-C2α, β, γ) PI3K-isoforms [11]. However, class I (PI, PI-4-P, PI-4,5-P_2_) and class II isoform (PI, PI-4-P) substrate specificities are different in terms of PI-4-5-P_2_, as class II does not use this PI, but class I does. To determine the category of PI3K isoform associating with CNGA1, solubilized rod outer segments (ROS) were immunoprecipitated (IP) with anti-CNGA1 antibody or non-immune IgG (control), and changes in PI3K activity were evaluated using PI-4,5-P_2_ as substrate. The results indicate that PI3K activity was associated with anti-CNGA1 IPs; no activity was associated with control IPs (Figure 1A-B). This experiment also suggested that as the substrate PI-4,5-P_2_ can only be utilized by class I PI3K, the PI3K associated with CNGA1 could be class I PI3K (either IA or IB), but could not be class II PI3K.

### Expression of PI3K isoforms in retinal rod photoreceptors

CNGA1 is expressed mainly in the plasma membranes of the rod photoreceptor outer segments [3]. To determine whether class I PI3K isoforms are expressed in rod outer segments (ROS), we immunoblotted ROS from dark- and light-adapted rats with anti-PI3Kα, anti-PI3Kβ, anti-PI3Kγ, anti-CNGA1, and anti-opsin antibodies. We found expression of all class I PI3K isoforms in ROS, irrespective of dark- or light-adaption (Figure 1D-F). The ROS specific proteins, CNGA1 and opsin, were used as internal controls (Figure 1C, G). The expression profile of PI3Kδ in ROS was not evaluated, as its expression is restricted exclusively to leukocytes [12].

### PI3Kγ is associated with the CNGA1 in light-adapted ROS

**Figure 2:**
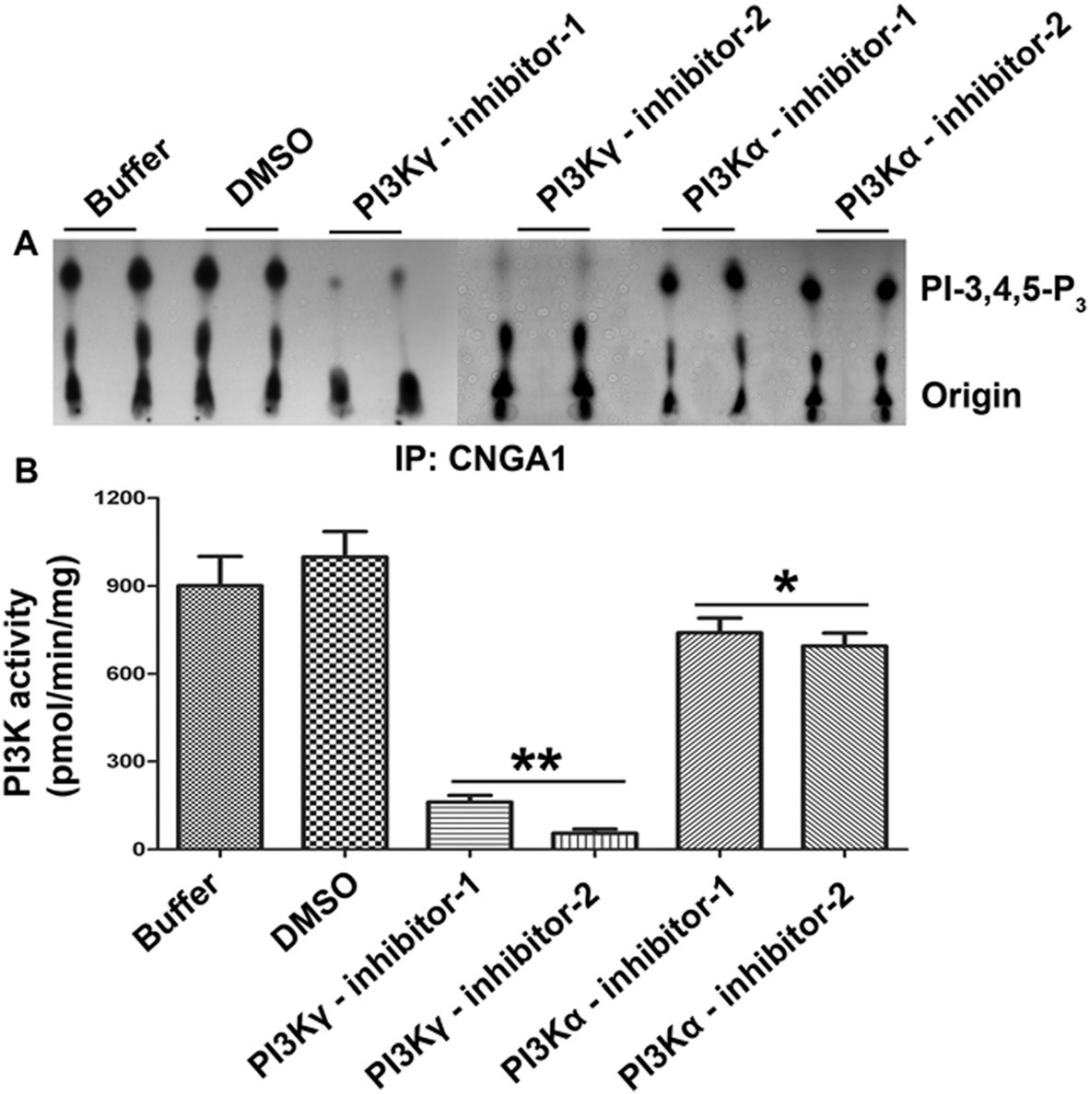
**PI3Kγ isoform predominantly associates with CNGA1.** Solubilized LROS was immunoprecipitated using anti-CNGA1 antibody and the washed immunoprecipitates were incubated with either buffer or DMSO or PI3Kγ inhibitor 1 and 2 or PI3Kα inhibitor 1 and 2 for 30 min. Then, the PI3K activity assay was carried out using PI-4,5-P_2_ and [γ^32^P]ATP as substrates **(A)**. The radioactive spots of PI-3,4,5-P_3_ were scraped from the TLC plate and counted using scintillation counter **(B)**. Data are mean ± *SD* (*n* = 4). *p < 0.05, **p < 0.001 (compared to buffer and DMSO control).

To establish the differential involvement of class IA or class IB PI3K associated with the CNGA1 in light-adapted ROS (LROS), we specifically immunoprecipitated (IP) CNGA1 with anti-CNGA1 antibody. The IPs were washed and incubated with either buffer or DMSO (vehicle to dissolve PI3K inhibitors), or PI3Kγ inhibitor 1 and 2 (1: AS605240, 8nM, 2: AS604850, 250 nM), or PI3Kα inhibitor 1 and 2 (1: PI-103, 8 nM, 2: PI3Kα inhibitor 2, 2 nM). We then carried out PI3K activity assays using PI-4,5-P_2_ as a substrate. Our results indicate that PI3K activity was inhibited by 85 ± 5% upon treatment with inhibitors of γ-isoform, while for the α-isoform activity was inhibited by 20 ± 5%, compared to either buffer or DMSO control (Figure 2). We found similar results with dark-adapted ROS (data not shown). These experiments largely highlight the association of the class IB PI3Kγ isoform with CNGA1.

### CNGA1-associated PI3Kγ activity is independent of dark and light conditions, as well as insulin receptor impairment in rods

**Figure 3:**
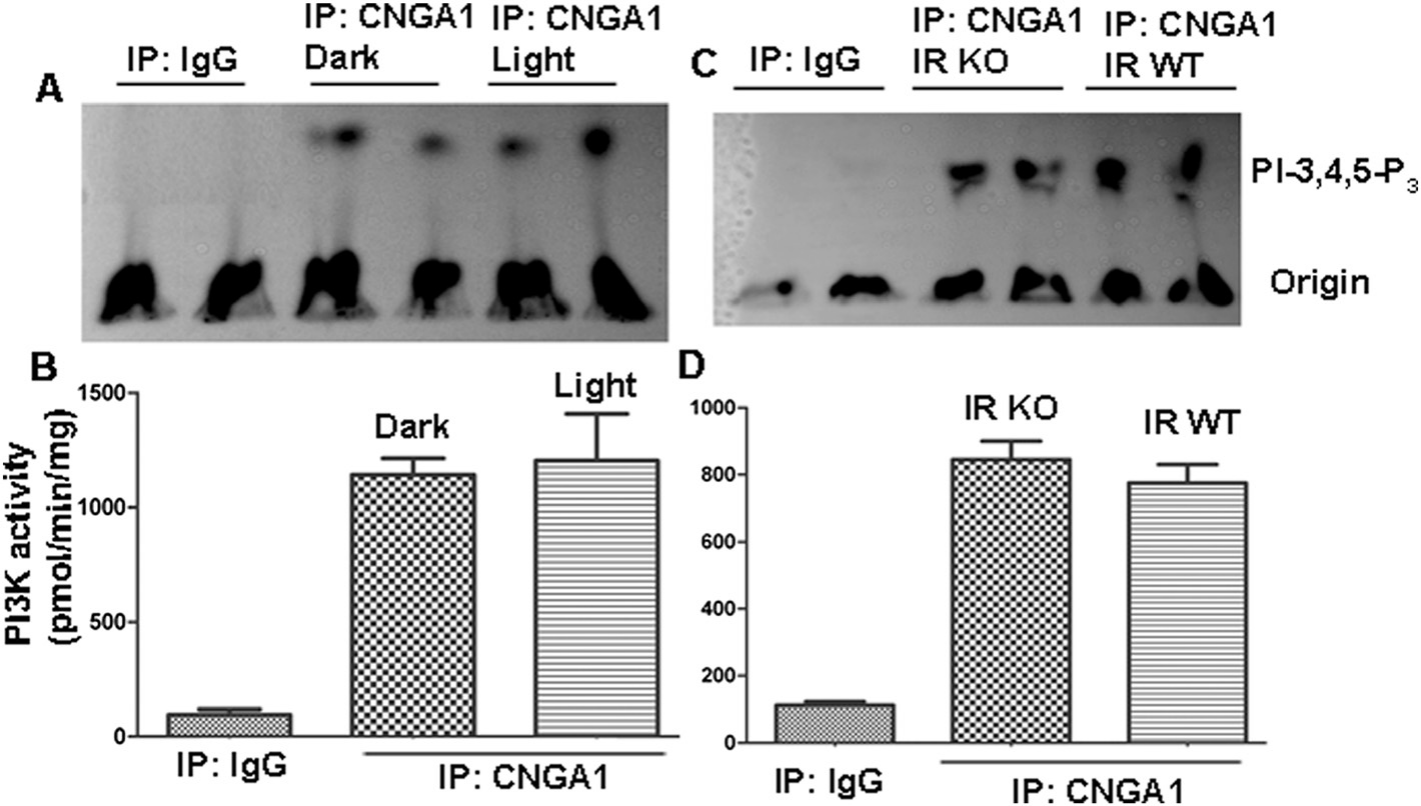
**PI3K activity associated with CNGA1 is independent of light and IR activation.** Solubilized ROS from either dark or light-adapted mice **(A)** or IR wild-type **(C)** or IR knockout **(C)** mice were immunoprecipitated with anti-CNGA1 antibody or non-immune IgG. PI3K activity was measured using PI-4,5-P_2_ and [γ^32^P]ATP as substrates. The radioactive spots of PI-3,4,5-P_3_ were scraped from the TLC plate and counted **(B, D)**. Data are mean ± *SD* (*n* = 3).

We previously reported a light-dependent association of PI3Kα with tyrosine-phosphorylated IR in rod photoreceptor outer segments [13]. To determine whether light has any effect on the association of PI3Kγ with CNGA1 and to further rule out the involvement of IR signaling in regulating PI3Kγ activation, we immunoprecipitated CNGA1 using anti-CNGA1antibody or normal IgG from the solubilized ROS of dark- and light-adapted mice, and from IR wild-type and IR knockout mice. PI3K activity was measured using PI-4,5-P_2_ as substrate. The results indicated that there was no significant difference between dark- and light-adapted (Figure 3A, B) or between IR wild-type and IR knockout (Figure 3C, D) mouse retinas, in terms of the association of PI3Kγ with CNGA1. These experiments clarify that, unlike activation of PI3Kα which is light- and receptor tyrosine kinase IR-dependent [14], the association of PI3Kγ with CNGA1 may be mediated through a simple protein-protein interaction between the RA and RLD domains.

### PI3Kγ interacts with CNGA1 through its RA domain

**Figure 4:**
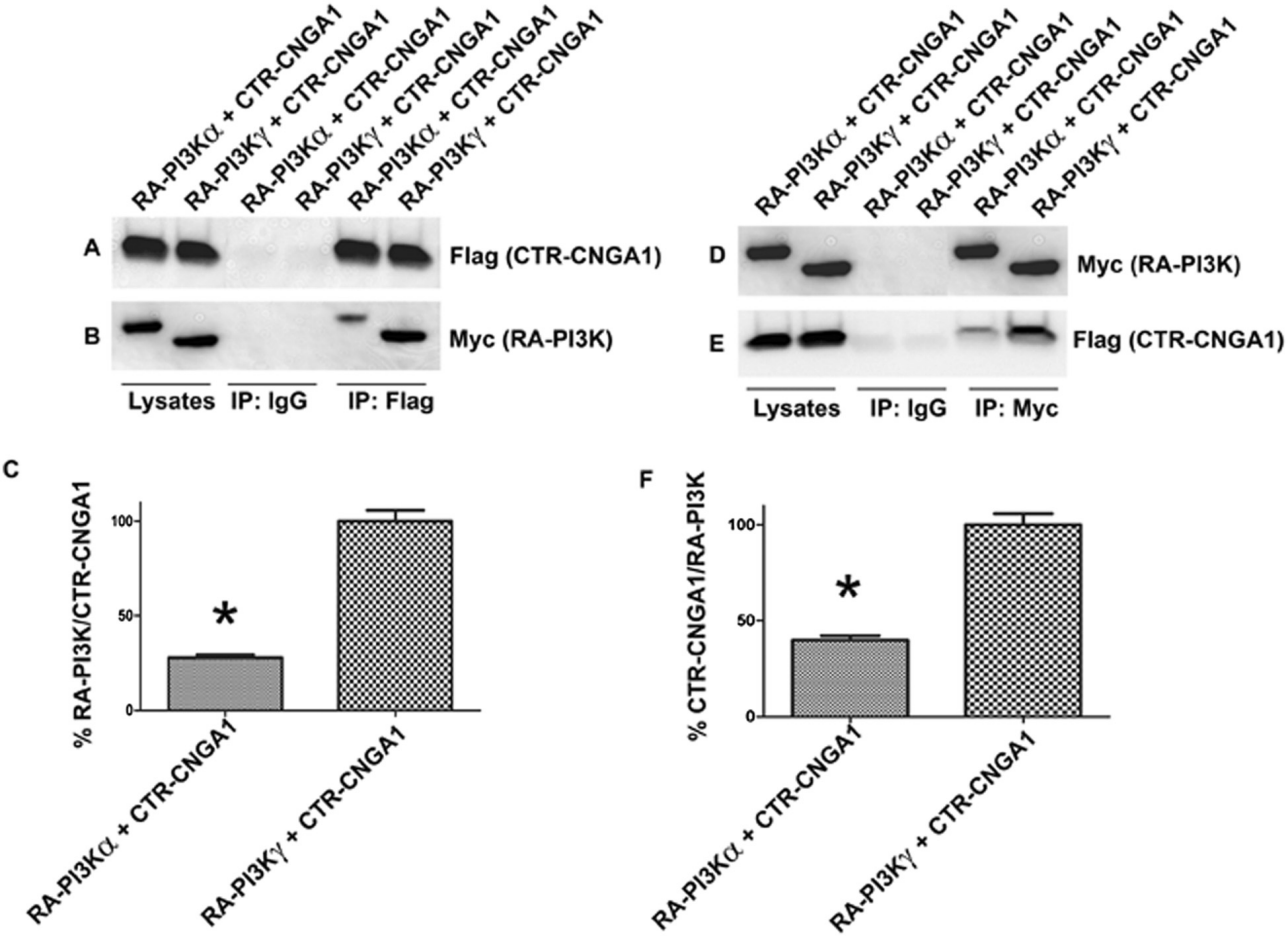
**PI3Kα and γ isoforms associate with CNGA1 through their RA domain.** The physical interaction of these domains was analyzed by reciprocal co-immunoprecipitations. The Flag-tagged RLD-CNGA1 domain and Myc-tagged PI3Kα and γ RA domains were transiently expressed in HEK-293 T cells. Expressed proteins were subjected to immunoprecipitation with either anti-Flag or anti-Myc antibodies or IgG. Anti-Flag and normal IgG IPs were subjected to immunoblot analysis with anti-Flag **(A)** and anti-Myc **(B)** antibodies along with Flag- and Myc-expressed protein lysates, whereas anti-Myc and normal IgG IPs were subjected to immunoblot analysis with anti-Myc **(D)** and anti-Flag **(E)** antibodies along with Myc- and Flag-expressed protein lysates. The relative binding was expressed as percentage of the RA-PI3K and RLD-CNGA1 and vice versa in each case **(C, F)**. Data are mean ± *SD*, *n* = 3, *p < 0.001.

The regions encompassing the RA domain of PI3Kα and PI3Kγ were cloned as in frame fusion with a Myc-tag into pcDNA3 vector and co-expressed in HEK-293 T cells along with a Flag-tagged cytoplasmic domain of CNGA1 (CTR) in pcDNA3 vector. The RA domain of PI3Kγ was expressed with an additional sequence at the N-terminal (sequence showing homology with RA-PI3Kα) as the RA domain (220-311, [2]) clone was not observed to be stably expressed in HEK-293 T cells under the present conditions. The reciprocal immunoprecipitations with Myc and Flag antibodies revealed that interaction between CNGA1 and PI3Kα or PI3Kγ is mediated through the interactions between complementary domains, the RA domain of PI3K and the RLD domain of CNGA1. The results also indicated that the RA domain of PI3Kγ interacts strongly with CTR-CNGA1, compared with the RA domain of PI3Kα (100% versus 30%, Figure 4).

**Figure 5:**
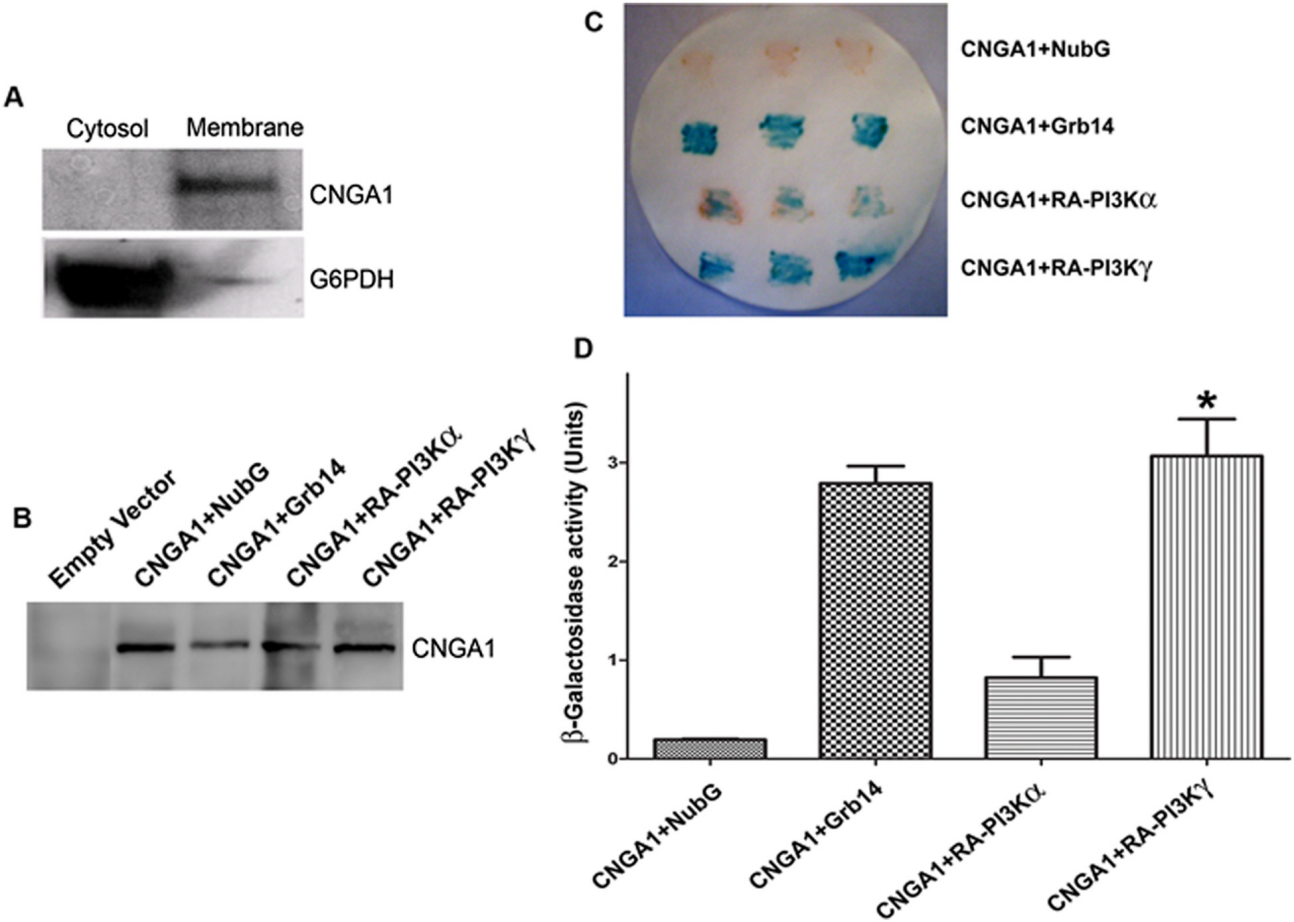
**PI3Kα and γ isoforms associate with CNGA1 through their RA domain, examined using membrane-based yeast two-hybrid analyses.** DSY-1 yeast cells co-expressing pDHB1-CNGA1 and pAlg5-NubG as negative control, CNGA1 + Grb14 as positive control, and CNGA1 + RA-PI3Kα and γ isoforms, respectively. The membrane and cytosolic fractions of yeast were subjected to immunoblot analysis with anti-CNGA1 and anti-G6PDH antibodies **(A)**. All other expressed proteins (either alone or co-expressed) were immunoblotted with anti-CNGA1 antibody **(B)**. Transformants were assayed for β-galactosidase assay by colony color by filter lift assay **(C)** or quantitative determination of β-galactosidase activity by the solution assay **(D)**. Data are mean ± *SD*, *n* = 6, *p < 0.001 (significance between RA-PI3Kγ compared to RA-PI3Kα).

The interaction between the RA domains of PI3Kα or PI3Kγ and CNGA1 were further evaluated by split ubiquitin-based membrane yeast two-hybrid analysis [10]. CNGA1 was observed to interact with RA-PI3Kγ more strongly than the RA-PI3Kα isoform. The strength of the interaction was quantified by liquid β-galactosidase activity assays. We observed that RA-PI3Kα derives the reaction to 25 ± 10%, compared to that of the RA-PI3Kγ isoform (Figure 5C and D). The protein expression of CNGA1 in the yeast was confirmed by immunoblotting (Figure 5B). The CNGA1 was observed to target properly to the membrane, as it was localized exclusively to the membrane fraction of the yeast and absent in the cytosolic fraction (Figure 5A). G6PDH was used as a marker for the cytoplasm (Figure 5A). The results further support that the RA domain of PI3Kγ interacts strongly with CTR-CNGA1, compared with the RA domain of PI3Kα.

### Effect of RA-PI3Kγ on channel activity

**Figure 6:**
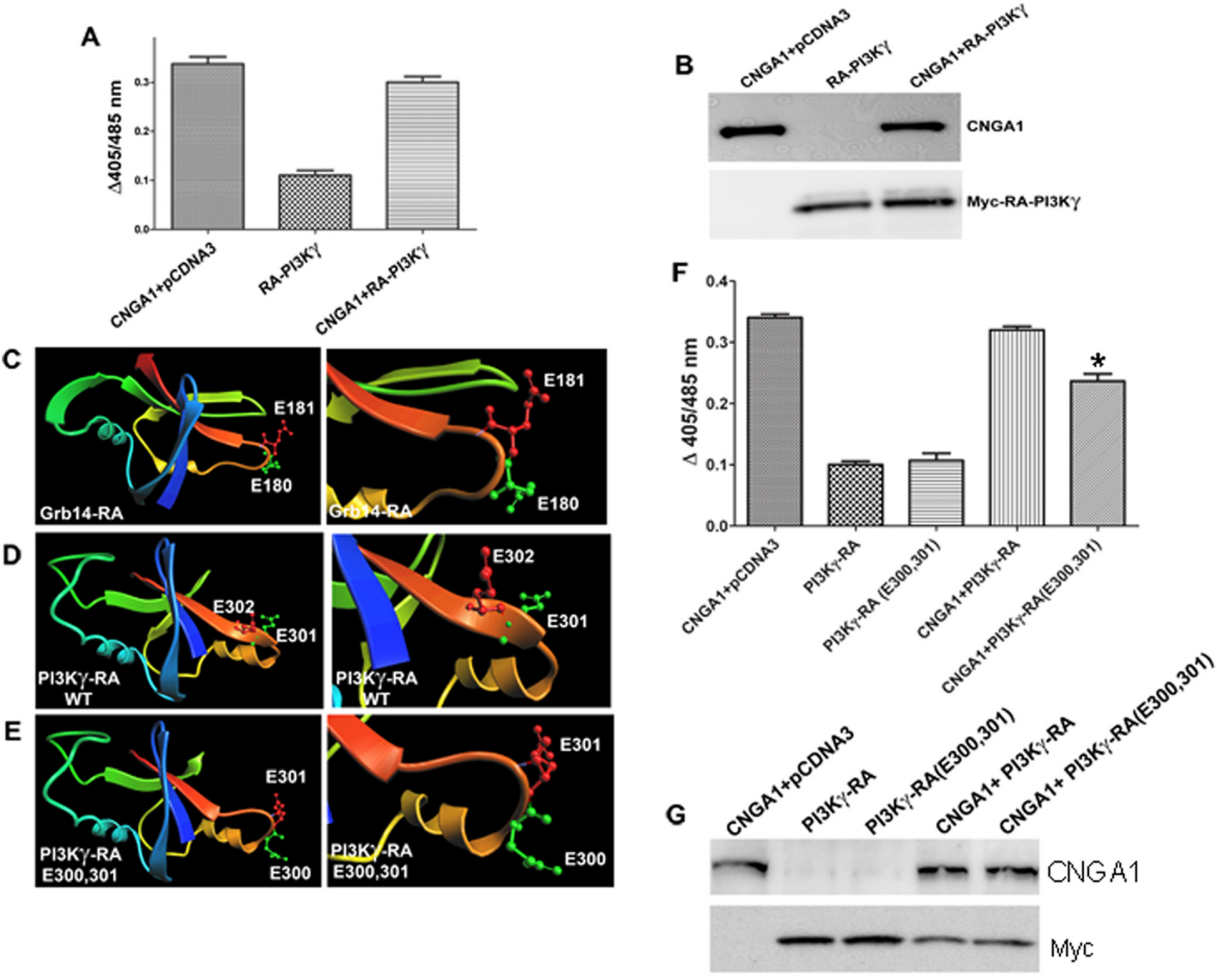
**Effect of RA-PI3Kγ on CNG channel activity.** HEK-293 T cells transfected with CNGA1 + empty plasmid (pCDNA3) or RA-PI3Kγ or CNGA1 + RA-PI3Kγ and channel activity was measured as a function of Ca^2+^ influx in response to cGMP stimulation **(A)**. The data are mean ± *SD*, *n* = 3 independent transfections. Protein expression in cell lysates was determined by immunoblotting with anti-CNGA1 and anti-Myc antibodies **(B)**. The RA domains of Grb14 **(C)**, PI3Kγ-wild-type **(D)**, and PI3Kγ-mutant (incorporation of two Glu in between Asn 299 and Gly 300; **E**) complete view and zoomed version are shown in each case. **Panel F** represents the channel activity measured as a function of Ca^2+^ influx in response to cGMP stimulation. Protein expression of PI3Kγ-wild-type and PI3Kγ-mutant alone or co-expressed along with CNGA1 in HEK-293 T cell lysates was determined by immunoblotting with anti-CNGA1 and anti-Myc antibodies **(G)**.

The Myc-tagged RA domain of PI3Kγ was co-expressed along with the CNGA1 in HEK-293 T cells, and its effect on CNGA1 channel activity was measured [10]. It was observed that co-expression with RA-PI3Kγ does not lead to an alteration in the channel activity. CNGA1 co-transfected with pCDNA3 and RA-PI3Kγ alone were used as controls (Figure 6A). To confirm that the lack of any effect on channel activity is not due to the absence of PI3Kγ expression, we immunoblotted the expressed proteins with anti-CNGA1 and anti-Myc antibodies. The results established comparable levels of CNGA1 and RA-PI3Kγ expression (Figure 6B).

We observed that the RA domain of CNGA1 exhibits considerable tertiary structural homology with the RA domain of Grb14, which is known to inhibit CNGA1 activity, as shown in Figure 6C. RA-Grb14 inhibits CNGA1 through the involvement of Glu,^180, 181^ which is present at the β-turn with a unique orientation [15]. Our *in silico* studies identified that, in RA-PI3Kγ, these Glu residues are present towards the β-sheet extension and their orientation does not allow them to interact adequately with the CNGA1 in a manner that would facilitate modulation of its activity (Figure 6D). These *in silico* predictions were verified by site-directed insertional mutagenesis, where two Glu residues were introduced at 300,301 positions between an Asn and a Gly residue (Figure 6E). This modification imparts PI3Kγ with the capability to inhibit CNGA1 significantly, as predicted from the model and as shown experimentally in the Figure 6F. To confirm the expression of proteins, immunoblotting was performed with anti-CNGA1 and anti-Myc antibodies. The results show comparable levels of protein expression under experimental conditions (Figure 6G). Together, these experiments suggest that, unlike Grb14, PI3Kγ may use CNGA1 as an adapter for activation, rather than playing a role in the modulation of CNG channel activity.

### Identification of the interaction constraints of CTR-CNGA1 and RA-PI3Kγ

**Figure 7:**
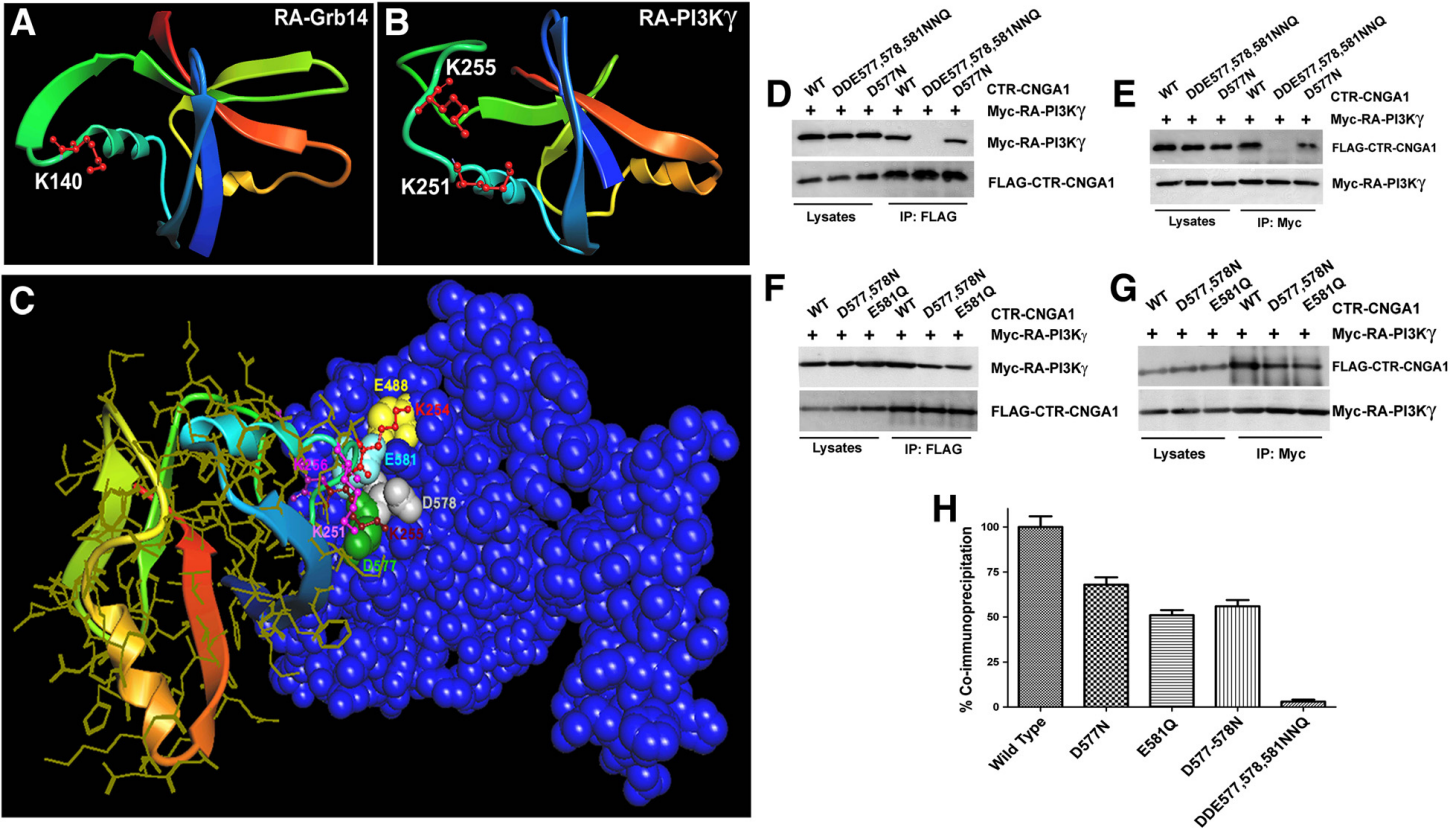
**Molecular determinants involved in RA-PI3Kγ interaction with CNGA1.** Ribbon-shaped model of the RA domain of Grb14 [UniProtKB Accession No. Q5ICW4] (106–192) constructed using the RA domain of Grb10 as a template (PDB ID: 3HK0). The interacting residues of RA-Grb14 **(A)** and RA-PI3Kγ **(B)** are highlighted as ball and stick (red) and labeled. The corresponding secondary structural motifs of the two structures are similarly colored. Complete view of the interaction between RA-PI3Kγ (ribbon, multicolored) and the cytoplasmic domain of CNGA1 (CPK, blue; **C**). The positively charged random coil element of RA-PI3Kγ with projecting Lys side chains (251 pink, 255 brown, 256 purple and 254 red; ball and stick, labelled) interacts with Asp^577^ (green space-filled and labeled), Asp578 (gray space-filled and labeled) and Glu581 (light blue, space-filled and labelled). Glu488 (yellow, space-filled and labeled) provides additional anchorage through the interaction with K254. The interacting residues of RA-PI3Kγ are highlighted as ball and stick and labeled, while the CNGA1 interacting residues are represented as space-filled structures and labeled. We investigated the interaction between RLD-CNGA1 and its mutant proteins by subjecting the HEK-293 T cell lysates expressing respective proteins to immunoprecipitation with anti-Flag antibody **(D)**, followed by immunoblotting and probing for RA-PI3Kγ with anti-Myc antibody **(D, F)** and vice versa **(E, G)**. Cell lysates were also loaded in each case for comparison. The interaction was quantified by densitometric analysis of the band intensities in immunoblots, performed in the linear range of detection, and normalized by the total amount of RLD-CNGA1 or RA-PI3Kγ immunoprecipitated in each case **(H)**. The relative values were expressed as percentage of the co-immunoprecipitation in wild-type proteins. Data are mean ± *SD*, *n* = 3, significance between each of the wild type RLD-CNGA1, D577N, E581Q, D577-578 N, and DDE 577,578, 581 NNQ *p < 0.001.

The RA domain of Grb14 interacts with CNGA1 and Ras proteins through the involvement of Lys140 residue [9,15] as shown (Figure 7A). The RA domain of PI3Kγ has also been shown to interact with Ras proteins through Lys residues (251, 255, 256). We observed that the domain is located on a similar tertiary structural fold as shown in Figure 7B. These observations suggest that RA-PI3Kγ and CTR-CNGA1 interactions are potentially mediated through Lys residues, which have protruded side chains and present a highly positively charged local environment to the protein motif. This region is more positively charged than the RA domain of Grb14, and its positive cloud is further enhanced by the K^254^ residue which is present in its vicinity (Figure 7C). We previously reported that RA-domain and RLD interactions are electrostatic [9] and require complementary charges on the interacting partner. The surface topology of the modeled CTR-CNGA1 [9,15] was probed for the distribution of negatively charged residues, which are easily accessible to the Lys residues of RA-PI3Kγ. The most negatively charged pocket on the surface of the CTR-CNGA1 suggested a potential sink for the most favorable interaction that may exist between these two domains. Molecular modeling revealed that K251, 255, and 256 forms a potential electrostatic interaction with the D577, D578, and E581 located on the surface of cytoplasmic region of CNGA1. Although E488 is not directly involved in interactions, it may still contribute to the formation of a negatively charged cloud. The complete view is shown in Figure 7C. The predictions made by molecular modeling were confirmed by site-directed mutagenesis, followed by the overexpression of the proteins in the HEK293 T cells (Figure 7D-G). Reciprocal co-immunoprecipitations of the tagged proteins revealed that the physical interaction was completely broken upon the mutation of all three residues: D577, D578, and E581 to N, N, and Q, respectively (Figure 7H). The individual or double mutation of the residues could not completely alleviate the interaction. Densitometric quantification revealed that the E581Q mutation can break the interaction by 50 ± 10 percent. D577N disrupted the interaction by 30 ± 10 percent, while an interaction disruption of 45 ± 10 percent was observed with a double mutation of D577, 578 N.

## Discussion

**Figure 8:**
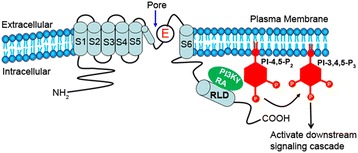
**Working model of CNGA1-associated PI3Kγ activation.** The CNG channel is localized to the plasma membrane of the rod photoreceptor cells. We propose that the RA-domain of PI3Kγ associates with the RLD-domain of CNGA1, and it will thereby have a close proximity to its substrates. PI3Kγ-generated PIs activate the downstream effectors and thereby regulate various aspects of photoreceptor functions.

Ras proteins form a direct contact with the PI3Kγ catalytic domain. These unique interactions are most likely also shared by the Ras/PI3Kα isoform [2]. Consistent with earlier findings, in the present study we found that the RA-domain of PI3Kγ associated with a Ras-like domain (RLD) in CNGA1 channel subunit. Our results further suggest that the RA-domain of PI3Kγ binds to the RLD region of CNGA1 significantly more strongly than does the RA-domain of PI3Kα. We also found that the RA-domain of PI3Kγ has no effect on channel activity, suggesting that PI3Kγ may tether to CNGA1 for its activation (Figure 8). It is interesting to note that the RA-domain of Grb14 can modulate the channel activity, but not the RA-domain of PI3Kγ. We previously reported that Grb14 competes with cGMP for the CNGA1 binding pocket, and electrostatically interacts with Arg^559^ through a negatively charged β-turn at its RA domain [15]. We showed that three Glu residues (180–182) in Grb14 are critical for electrostatic interaction with the cGMP binding pocket and the resultant inhibition [15]. Tertiary structural analysis revealed that the RA domain of Grb14 shows a 50-60% structural similarity with the RA domain of PI3Kγ, with the position of residues and arrangement of most secondary structural motifs conserved. The RA domain of PI3Kα is not significantly similar in structure to the RA domains of either PI3Kγ or Grb14.

Examination of the RA domains in PI3Kγ, PI3Kα, and Grb14 suggests a molecular divergence among the RA domains in intracellular signaling proteins. We attributed this difference between RA-PI3Kγ and RA-Grb14’s inhibitory effects on the channel to the orientation of the Glu residues in the β-turn. We confirmed this hypothesis by site-directed insertional mutagenesis in which two Glu residues were introduced at the 300,301 positions between an Asn and a Gly residue, and found a significant inhibition of channel activity by RA-PI3Kγ.

The fraction of the total PI3Kγ bound to CNGA1 in photoreceptors is unknown. Nevertheless, it must constitute an important proportion of the total pool, as the alternative Ras-mediated pathway is insufficient to drive membrane translocation of PI3Kγ, a necessity for its activation [16]. Alternatively, this binding might complement the Ras-PI3Kγ interaction pathway by accepting the allosterically activated PI3K from Ras and enabling it to stay bound at the membrane. The interaction of CNGA1 with PI3Kγ also indicates that CNGA1 may contribute towards the existence of an alternative pathway to Ras activation of PI3Kγ, strengthened by the fact that both CNGA1 and Ras proteins are nucleotide binding proteins showing similar structural folds. The relative contribution of these controlling mechanisms, as well as the fraction of the PI3Kγ participating in each of the regulatory interactions, is yet to be determined *in vivo*.

The crystal structure of PI3Kγ in complex with Ras has revealed that K^251, 254, 255,^ and ^256^ are critical residues for establishing an interaction with Ras [2]. Lys^140^ of Grb10 [17] and Grb14 [15] have been shown to be involved in the interaction of the RA domain with Ras proteins and the Ras-like domain of CNGA1. Lys^140^ of Grb14 corresponds to the Lys^251^and Lys^255^ of the RA-PI3Kγ, but is present amongst a highly positively charged cloud along the β-turn, imparting it with more leverage to interact and associate with proteins carrying negatively charged surface pockets. These types of electrostatic interactions comprise the basis of PI3Kγ’s interaction with Ras proteins [2]. These interactions may also be a good model for the weak or potentially transient interaction of RA-PI3Kα with CNGA1. In addition, these interactions might contribute to conformational changes in PI3Kγ, similar to those caused by Ras protein binding [2] and leading to altered PI3Kγ activity and membrane recruitment. The structural folds of the Ras-associating domain of PI3Kγ are also similar to Raf and RalGDS, two other effectors of Ras proteins [18,19].

The phospholipids have been shown to play significant roles in the negative modulation of CNG channels [20-23]. Even though PI3Kγ may not have a direct effect on CNG channel modulation, PI3Kγ-generated PI-3,4,5-P_3_ may indirectly influence the channel activity. Accordingly, PI-3,4,5-P3 has been previously shown to inhibit the action of olfactory [5] and cone CNG [7] channels. Our studies thus suggest a functional non-canonical PI3Kγ activation in retinal rod photoreceptor cells.

## Materials and methods

### Cell lines and culture conditions

HEK-293 T cells were maintained at 37°C in DMEM medium containing 10% (v/v) FBS. Approximately 2.5 × 10^5^ cells were seeded in each 60-mm culture dish 12–18 h before transfection. Calcium phosphate-mediated DNA transfection was performed using each of the plasmids containing the cDNA of interest [24]. Cells were harvested for experiments ~48 h post-transfection.

### Plasmids and DNA

The regions encompassing the RA domain of PI3Kα (Uniprot id. P42336, residues 132–314) and PI3Kγ isoform (P48736, residues 141–311; Open Biosystems, Huntsville, AL, USA) were amplified from their respective full-length PI3Ks and cloned into Myc-tagged pCDNA3 vector with Bam H1 and Xho1 sites. The CNGA1 channel antibodies were a kind gift from Dr. Robert Molday, University of British Columbia (Canada). The Myc-tagged CNGA1 construct was generated from the amplification of pCDNA3-CNGA1 with sense (GAA TTC ACC ATG GAG CAA AAA CTC ATC TCA GAA GAG GAG GAT CTG ATG AAG AAA GTG ATT ATC AAT ACA TGG CAC ) and antisense (CTC GAG TCA GTC CTG TGT AGA GTC TGT GGG CCC ACT TTC) primers. The NH_2_-terminal FLAG-tagged C-terminal region of CNGA1 (CTR-CNGA1,residues 483–690) was cloned into pCDNA3 using sense (GAA TTC ACC ATG GAT TAC AAG GAT GAC GAC GAT AAG GCT TGG TCT GTT GGT GGAG) and antisense (CTC GAG TCA GTC CTG TGT AGA GTC TGT GGG CCC ACT TTC) primers. The cDNA encoding the full-length CNGA1 was cloned into the *NcoI* site of pDHB1 membrane-based yeast two-hybrid bait vector. Full-length Grb14, RA-PI3Kγ, and RA-PI3Kα isoforms were cloned as *BamH1/Sal1* into pDL2-Nx prey vector. The positive (pMBV-Alg5 and pAlg5-NubI) and negative (pMBV-Alg5 and pAlg5-NubG) plasmids were obtained from Dualsystems Biotech AG (Switzerland). The membrane yeast two-hybrid assay was carried out using the Dual hybrid kit (Dualsystems Biotech AG, Switzerland). The membrane yeast two-hybrid screen was used in the *S. cerevisiae* strains DSY-1. Site-directed mutagenesis (SDM) was carried out according to the method described earlier [25]. The primers used for SDM were PI3Kγ-RA (E 300, 301; sense: TGC CTC AAG AAC GAA GAA GGA GAA GAG ATT; antisense AAT CTC TTC TCC TTC TTC GTT CTT GAG GCA), CTR-CNGA1(DDE 577, 578, 581 NNQ; sense: TCA AAA AAT ACC CTC ATG CAA GCT CTA ACT; antisense: AGT TAG AGC TTG CAT GAG GTT ATT TTT TGA), CTR-CNGA1 (D577N; sense: CTC TCA AAA AAT GAC CTC ATG GAA GCT CTA; antisense: TAG AGC TTC CAT GAG GTC ATT TTT TGA GAG), CTR-CNGA1 (D577,578 N; sense: CTC TCA AAA AAT AAC CTC ATG GAA GCT CTA; antisense: TAG AGC TTC CAT GAG GTT ATT TTT TGA GAG), CTR-CNGA1 (E581Q; sense: AAA GAT GAC CTC ATG CAA GCT CTA ACT GAG; antisense: CTC AGT TAG AGC TTG CAT GAG GTC ATC TTT). After sequencing, the mutant cDNAs were excised from the sequencing vector and cloned into pCDNA3 mammalian expression vector.

### Animals

All animal work was performed in strict accordance with *the NIH Guide for the Care Use of Laboratory Animals*, and the Association for Research in Vision and Ophthalmology on the Use of Animals in Vision Research. All protocols were approved by the IACUC of the University of Oklahoma Health Sciences Center and the Dean McGee Eye Institute. The generation of rod-specific conditional IR knockout mice has been reported previously [26]. A breeding colony of Albino Sprague–Dawley rats is maintained in our vivarium in cyclic light (12 h on/ off; ~300 lux). Experiments were carried out on male and female rats and mice.

### Membrane-based yeast two-hybrid assay

Yeast transformations were performed using lithium acetate [25]. We used the split ubiquitin system to genetically investigate interactions between membrane proteins [27,28] to probe the possible intercommunications between full-length CNGA1 and either of the full-length Grb14, RA-PI3Kα, or RA-PI3Kγ, using the transcription factor protein-LexA-VP16 (PLV) as the reporter molecule. The filter paper β-galactosidase assay for the detection of interactions and liquid culture assay for their quantification was performed as described previously [29].

### PI3K activity assays

Mouse and rat photoreceptor outer segments (ROS) were prepared as described previously [13]. Enzyme assays were performed on solubilized ROS, essentially as described previously [30]. Briefly, assays were performed directly on anti-CNGA1 immunoprecipitates in 50 μl of the reaction mixture containing 0.2 mg/ml PI-4, 5-P_2_, 50 μM ATP, 0.2 μCi [γ^32^P] ATP (Perkin Elmer, MA, USA), 5 mM MgCl_2_, and 10 mM HEPES buffer (pH 7.5). The reaction was performed for 30 min at 25°C and was stopped by the addition of 100 μl of 1 N HCl followed by 200 μl chloroform-methanol (1:1 v/v). Lipids were extracted and resolved on TLC plates (silica gel 60) with a solvent system of 2-propanol/2 M acetic acid (65/35, v/v). The plates were coated in 1% (w/v) potassium oxalate in 50% (v/v) methanol and then baked in the oven at 100°C for 1 h before use. TLC plates were exposed to X-ray film overnight at −70°C. Radioactive lipids were scraped and quantified by liquid scintillation counting.

### Assessment of the channel activity by ratiometric measurement of [Ca^2+^]_i_

The fluorescent indicator Indo-1/AM was used to monitor Ca^2+^ influx through the CNGA1 channels in cell suspensions. The assays were performed as described [10,15] using a spectrofluorometer (Fluostar Omega, BMG lab tech GmBH, Offenburg, Germany). This assay was designed to determine CNG channel activity in cell populations (2 × 10^6^) in response to 8-pCPT-cGMP stimulation. Briefly, cells (36–48 h post-transfection) were harvested with cell dissociation medium (Invitrogen, Carlsbad, CA), washed with the extracellular solution (ECS; 140 mM NaCl, 5 mM KCl, 1 mM MgCl_2_, 1.8 mM CaCl_2_, 10 mM glucose, 15 mM HEPES, pH 7.4), and incubated with 2 μM Indo-1/AM (Sigma-Aldrich) in ECS in the presence of 0.05% Pluronic F-127 (Invitrogen, Carlsbad, CA) for 40 min at room temperature. Then, the cells were washed three times with ECS and resuspended in ECS (1 × 10^6^/mL). Ca^2+^ influx in response to 8-pCPT-cGMP was determined by ratiometric measurement, which represents the free intracellular Ca^2+^ concentration. Changes of intracellular Ca^2+^ concentration were expressed as a ∆405/485 ratio.

### Molecular modeling studies

The protein tertiary structural modeling was done using the MODELLER program [31]. Structural manipulations, surface topology studies, and graphical representations were performed using Arguslab (Thompson, Planaria software), VMD [32], and DaliLite v3 [33]. The extensive energy minimization was done using Deep View [34]. Structural constraints and prediction quality of the modeled structures were evaluated using WHATIF [35] and PROCHECK [36].

### Statistical methods

Data were analyzed and graphed using Graphpad Prism software (GraphPad Software, San Diego, CA). The data were expressed as the mean ± *SD* and compared by Student’s *t* test for unpaired data. The critical level of significance was set at *p* < 0.05.
